# Consequences of Morphology on Molecularly Imprinted Polymer-Ligand Recognition

**DOI:** 10.3390/ijms14011207

**Published:** 2013-01-09

**Authors:** Annika M. Rosengren, Björn C. G. Karlsson, Ian A. Nicholls

**Affiliations:** 1 Bioorganic & Biophysical Chemistry Laboratory, Linnæus University Centre for Biomaterials Chemistry, Linnæus University, SE-391 82 Kalmar, Sweden; E-Mails: annika.rosengren@lnu.se (A.M.R.); bjorn.karlsson@lnu.se (B.C.G.K.); 2 Department of Chemistry, Uppsala University, PO Box 576, SE-751 23 Uppsala, Sweden

**Keywords:** molecularly imprinted polymer, MIP, morphology, BET, surface area, warfarin

## Abstract

The relationship between molecularly imprinted polymer (MIP) morphology and template-rebinding over a series of warfarin-imprinted methacrylic acid *co*(ethylene dimethacrylate) polymers has been explored. Detailed investigations of the nature of template recognition revealed that an optimal template binding was obtained with polymers possessing a narrow population of pores (~3–4 nm) in the mesopore size range. Importantly, the warfarin-polymer rebinding analyses suggest strategies for regulating ligand binding capacity and specificity through variation of the degree of cross-linking, where polymers prepared with a lower degree of cross-linking afford higher capacity though non-specific in character. In contrast, the co-existence of specific and non-specific binding was found in conjunction with higher degrees of cross-linking and resultant mesoand macropore size distributions.

## 1. Introduction

The molecular imprinting technique provides a means for creating robust polymeric recognition materials with predetermined ligand selectivities [1–4]. Previous studies have highlighted the large number of factors that influence polymer-ligand recognition [5–8]. Over the past decade significant inroads have been made towards elucidating the nature of the molecular level mechanisms governing the recognition characteristics of these materials. Computational and spectroscopic strategies, in particular, have provided valuable insights, that have in-turn been used in efforts to establish rational design strategies [9–12]. While our understanding of the impact of molecular-level factors related to the pre-polymerization stage on subsequent polymer-ligand recognition has developed, the relationships between the nature of molecular imprinting polymerization mixtures and commensurate polymer morphologies has been relatively unexplored. In this area only a handful of reports have appeared to date, in particular seminal work by Sellergren and Shea [5]. While empirical evidence has suggested that ligand selective recognition, in particular binding kinetics are influenced by rates of mass transfer [13,14], descriptive correlations between polymer composition and morphology, and morphology and ligand binding remain elusive. More recent examination of the nature of binding to (non-imprinted) particles of various sizes highlighted the significance of particle size and surface area on recognition behavior [15]. The relationship between the influence of degree of cross-linking on polymer morphology and template recognition remains elusive.

In previous studies, warfarin-imprinted polymers were designed based upon selection of candidate functional monomers derived from a series of molecular dynamics and NMR studies of potential pre-polymerization mixtures. Initial synthesis and template recognition of candidate polymers identified a methacrylic acid—ethylene dimethacrylate co-polymer system—as the most promising [16]. Interest in warfarin selective materials stems from the use of this coumarin derivative as an anticoagulant in the treatment of thrombotic disorders such as myocardial infarction and stroke [17,18]. Several of the polymers in this study were initially developed for a larger study aimed at establishing methods to facilitate the direct measurement of warfarin in blood plasma using fluorescence spectroscopic techniques [19,20], and the correlation of warfarin distribution and its effect on blood coagulation [21,22].

**Chart 1:**
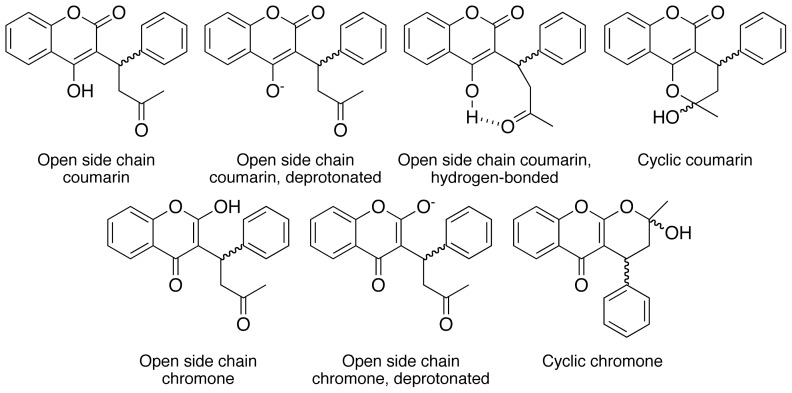
Structures of warfarin isomers.

Warfarin’s structural diversity poses a challenge for understanding its biological action due to its capacity to undergo environment-dependent isomerization, Chart 1, where the position of equilibrium between isomers is dependent upon the nature of the solvent and pH (molecular environment) [23–26]. It was observed that variation in the relative stoichiometry of the functional monomer and cross-linker used in this polymer induced observable differences in morphology and binding characteristics and suggested the possibility of controlling warfarin binding capacity or specificity when varying the degree of cross-linking. This observation motivated a more comprehensive study in an attempt to investigate possible correlations between morphology and template (warfarin) recognition behavior. The results arising from this study indicate that warfarin (template) recognition can be steered through manipulation of polymer morphology.

## 2. Results and Discussion

Molecularly imprinted polymers (MIPs) have become established as materials for selective recognition in an increasing number of analytical applications, e.g., solid phase extraction matrices, sensor recognition elements, and stationary phases for chromatography and electrophoresis [4]. Accordingly, approaches for optimizing the selectivity and capacity of these materials are of significant interest. Previously observed indications [16] of variation in morphologies and template-recognition as a function of the degree of cross-linking of warfarin imprinted methacrylic acid-ethylene dimethacrylate co-polymers motivated a more thorough investigation.

A series of five warfarin MIPs and their corresponding non-imprinted reference polymers (REFs) was prepared with stoichiometries as presented in Table 1 (see experimental section). The concentration of methacrylic acid employed was varied while maintaining the template and cross-linker concentrations constant. Accordingly, the template-functional monomer stoichiometries varied over the range 1:2 to 1:40, and the functional monomer cross-linker stoichiometries ≈1:28 to 1:4. Polymer monoliths were ground and sieved to afford particles with diameters of 25–50 μm.

**Figure 1:**
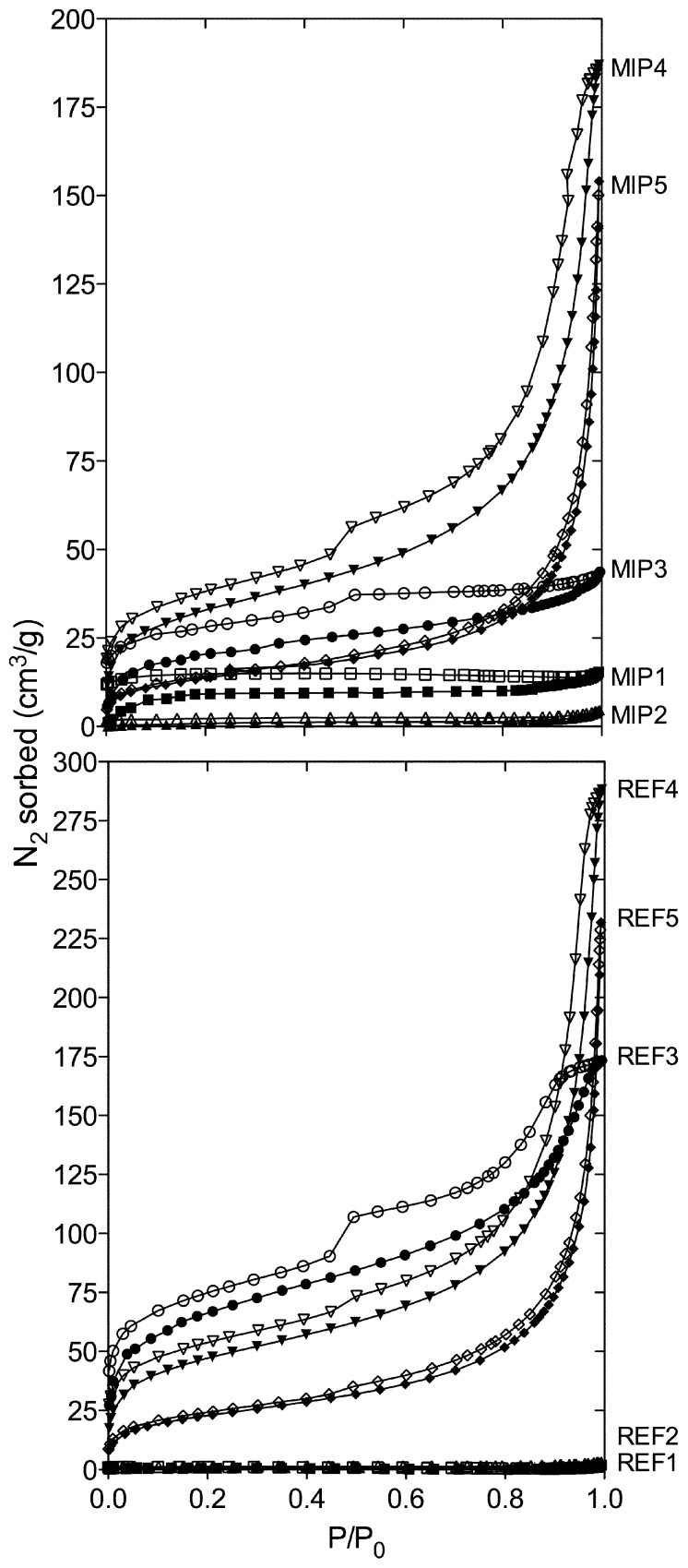
Nitrogen sorption isotherms obtained from measurements on the molecularly imprinted polymer (MIP) (top) and the reference polymers (REFs) (bottom). Where, closed symbols represent nitrogen adsorption and open symbols represent nitrogen desorption: MIP/REF1 (▪□), MIP/REF2 (▴▵), MIP/REF3 (●○), MIP/REF4 (▾▿) and MIP/REF5 (◆◇).

Polymers were subjected to nitrogen sorption studies. The resultant isotherms demonstrated variation with respect to both the degree of cross-linking and the total pore volume, Figure 1. In particular, it was apparent that variation in the molar ratio of methacrylic acid (MAA) and ethylene dimethacrylate (EDMA) had a fundamental impact upon the morphology of the resultant polymers, as seen by the similarities in isotherm profiles for the imprinted and corresponding non-imprinted materials. This in itself highlights the importance of MAA and the degree of cross-linking on morphology. The low surface areas and total pore volumes observed in polymers associated with a higher molar ratio of EDMA, MIP/REF1 and MIP/REF2, is indicative of the lack of pores in these materials. As anticipated, decreasing the molar ratio of EDMA resulted in more porous materials, as evidenced by a change in the isotherm profile at *P*/*P*_0_ > 0.3 and the existence of a hysteresis loop in the nitrogen desorption for MIP/REF3, MIP/REF4 and MIP/REF5, Figure 1.

**Figure 2:**
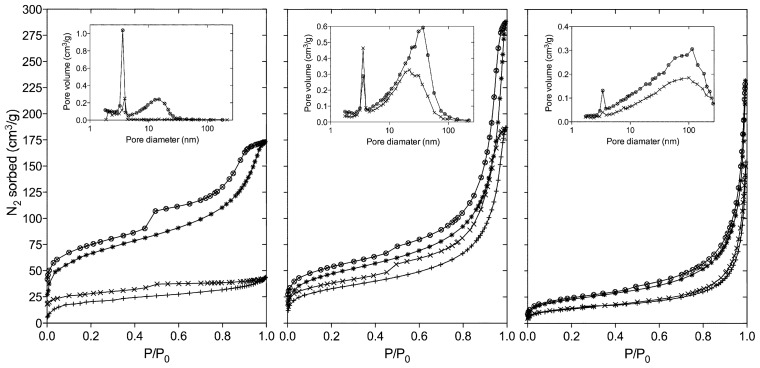
Nitrogen adsorptions (ads) and desorption (des) isotherms obtained from measurements on MIP (ads: +, des: ×) and REF polymers (ads: *, des: ⊗). BJH pore size distributions (inserted) for MIP/REF3 (**left**), MIP/REF4 (**middle**) and MIP/REF5 (**right**).

Although the nitrogen isothermal sorption profiles for the imprinted and corresponding REF polymers bear apparent similarities, a more detailed comparison within each MIP-REF polymer pair afforded further insights, Figure 2. A BJH pore size distribution plot produced from the desorption isotherms demonstrated the presence of differences in pore size distributions between the MIP and corresponding REF materials, Table 2. From these analyses two pore size distributions were found where the first a narrow distribution at 3.4–3.9 nm and the second a broader distribution comprised of larger pores in the mesopore (2–50 nm) to the macropore (>50 nm) ranges. Polymer system 3 (MIP3/REF3) was of particular interest as comparison of the MIP and the corresponding REF polymers showed the most significant differences in morphology within this pair, as also reflected in differences in surface area and total pore volume.

**Figure 3:**
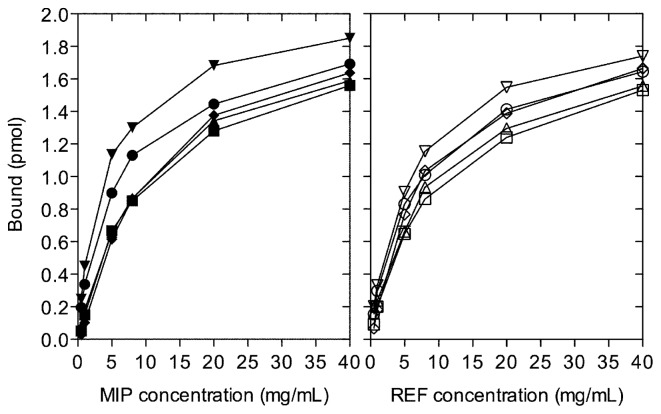
Radio-ligand polymer titration data derived from template rebinding in toluene at 293 K. MIP (1: ■, 2: ▲, 3: ●, 4: ▼ and 5: ◆) and REF (1: □, 2: △,3: ○, 4: ▽ and 5: ◇). All experiments were performed in triplicate and values are presented as mean standard error of the mean (≤ 0.03 pmol).

To be able to correlate the observed physical characteristics of the polymers with the capacity to bind warfarin, a series of polymer titration studies was performed using a radio-ligand binding assay, Figure 3. The calculation of the polymer concentration required to achieve 50% rebinding of template (PC_50_) made it possible to compare the binding capacities of the different polymers. Since the morphology of the polymers analyzed was found to vary significantly, a normalization of the binding of warfarin (pmol/m^2^) was introduced in order to allow a reasonable comparison of the polymer binding capacity. As it was considered probable that the warfarin rebinding would be proportional to the total surface area of the polymer, BET and Langmuir surface areas were determined from the nitrogen adsorption isotherms, Table 3. It is noteworthy that the surface areas derived through the use of the two different analysis methods employed in this study resulted in similar surface areas. Even so, as it is probable that the nitrogen adsorbs to the surface as a multilayer the BET surface area was used in the normalization of warfarin binding.

**Figure 4:**
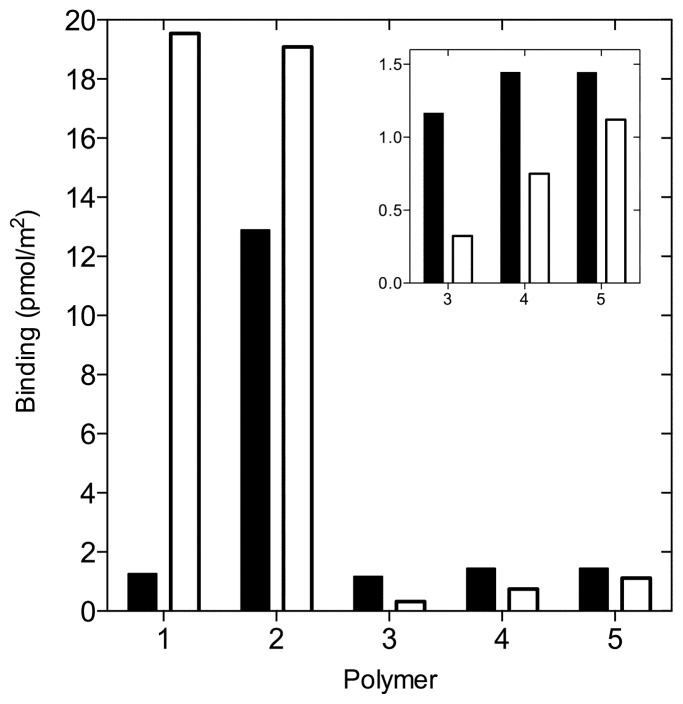
Polymer-warfarin binding data. Filled bars correspond to warfarin binding to the MIP whereas open bars correspond to warfarin binding to the REF polymer. Normalized binding data (PC_50_) is scaled with the corresponding swelling corrected polymer surface area, A_sw_ (Equation 2).

Interestingly, this treatment of the binding data revealed that different mechanisms steer binding to the polymers studied, and that these mechanisms are apparently highly dependent upon the surface area and porosity, Figure 4. In the case of binding to the non-porous polymers, polymer systems 1 and 2, only non-specific binding of warfarin to the polymer was observed. In these systems, the binding capacities of the REF polymers were found to be higher than for the MIPs. One explanation for this behavior can be found when considering the polymer composition. We suggest that using a too low MAA-template (warfarin) molar ratio in the MIP pre-polymerization mixture results not only in insufficient complexation of template but also leads to a polymer which will demonstrate a lower degree of crosslinking and in turn a higher swelling capacity [27]. In this study, based upon their observed swelling capacities, Table 3, polymers prepared with high molar ratios of EDMA, systems 1 and 2, demonstrated a low degree of crosslinking. This is in contrast to warfarin binding to the REF polymers in which MAA accessibility is not as limited, leading to a higher portion of non-specific binding to the REF polymers.

Warfarin rebinding to the more porous polymers (polymer systems 3–5) demonstrated a marked decrease in binding capacities when compared to systems 1 and 2. Interestingly, for these polymers warfarin rebinding was found to be higher for the MIPs compared to the REF polymers with an optimum binding difference observed for system 3. Since this polymer system demonstrated the largest difference in surface area and porosity, we suggest that warfarin binding sites derived from the molecular imprinting process typically have a diameter in the size range ~3–4 nm (warfarin has a diameter of ~1.0 nm) and that the homogeneous pore size distribution of MIP3 is induced by the presence of the template during polymerization in combination with a favorable stoichiometric relationship to MAA and EDMA. Finally, decreasing the molar ratio of EDMA further led to increased non-specific binding to REF4 and REF5 polymers, and a commensurate increase in polymer porosity. It is interesting to note that using a EDMA:MAA molar ratio 27.5–11.0 leads to approximately constant warfarin rebinding to the REF polymers whereas the molar range 4.6–1.4 leads to approximately constant rebinding to the MIPs.

## 3. Experimental Section

### 3.1. Chemicals

Racemic warfarin (3-(α-acetonylbenzyl)-4-hydroxycoumarin, >98%) was purchased from Sigma-Aldrich (St. Louis, MO, USA). Methacrylic acid (MAA) (KeboLab, Täby, Sweden) was distilled prior to use. Ethylene dimethacrylate (EDMA) (98%, Sigma-Aldrich, Steinheim, Germany) was extracted with NaOH (*aq*, 1.0 M), dried over magnesium sulfate and stored at 257 K under nitrogen. 2,2′-azo-bis(isobutyronitrile) (AIBN) was obtained from Janssen Chimica (Geel, Belgium). [^3^H]-(*R,S*)-warfarin (specific activity 13.7 Ci mmol^−1^) was from Moravek Biochemicals Inc. (Brea, CA, USA). All solvents were of analytical grade and were used as received.

### 3.2. Polymer Syntheses

Warfarin, MAA, EDMA and AIBN were dissolved in the porogen chloroform (CHCl_3_) in 50 mL test tubes (KIMAX-KIMBLE, 150 × 25 mm with screw caps and Teflon faced rubber liners), Table 1. The mixtures were cooled on ice and sparged with N_2_ for 20 min to remove dissolved oxygen. Tubes were subsequently placed on a low profile roller (ROL230, STOVALL, Green-Boro, NC, USA) before being irradiated with UV-light (365 nm) at 10 °C for 24 h using a BLAK-RAY B 100 AP inspection lamp. The resultant bulk polymers were manually ground and initially dry-sieved through a 50 μm sieve. Polymer particles were then wet-sieved through a 25 μm sieve using 600 mL of acetone. The fine particles were subsequently removed by repeated sedimentation from acetone (3 × 250 mL, 15 min). Polymer particles (~3 g) were slurry packed into HPLC columns then washed with a series of mixtures of methanol and acetic acid (4:1; *v*/*v*, 450 mL), ethanol (100 mL), a mixture of methanol, sodium hydroxide (*aq*, 5 M) and water (5:2:3; *v*/*v*/*v*, 200 mL), methanol (100 mL), a mixture of methanol, acetic acid and water (18:1:1; *v*/*v*/*v*, 200 mL), methanol (300 mL) and finally acetone (50 mL). The polymers were emptied from the column, dried for 24 h at 323 K and then stored at room temperature in a desiccator until further use. Corresponding non-imprinted, reference polymers (REF 1 –REF 5) were prepared identically, though in the absence of the template structure warfarin.

### 3.3. Polymer Surface Area and Porosity Measurements

Prior to nitrogen sorption measurements, samples were degassed at 323 K for 24 h to remove adsorbed gases and moisture. BET [28] and Langmuir [29] surface areas for the polymers were calculated from the adsorption data using 0.162 nm^2^ as the molecular cross-sectional area [30] for adsorbed nitrogen molecules. The BJH [31] method was applied to calculate the pore size distribution from the adsorption and desorption branches of the isotherms. All measurements were performed on an ASAP 2004 instrument (Micromeritics, Norcross, GA, USA) using 0.14–0.15 g polymer at 77 K.

### 3.4. Radioligand Binding Studies

Polymer titrations were performed at 293 K in toluene, which was used instead of chloroform as the rebinding medium since chloroform is an efficient quencher and interferes with the scintillation counting process. Binding to both the imprinted and the reference polymer was examined. The amount of polymer in each sample was in the range from 0.5 to 40 mg. [^3^H]-(*R*,*S*)-warfarin (2.2 pmol) and toluene were added to a total volume of 1.0 mL. Each experiment was performed in triplicate. The tubes were incubated (3 h) on a rocking table and then centrifuged (8000× *g*, 5 min). The supernatant (600 μL) was removed from each tube, mixed with scintillation cocktail (Beckman Ready Safe™, 2 mL) and counted (2 min, Packard Tri-Carb 2100TR liquid scintillation counter).

### 3.5. Swelling Studies

The volume of dry polymer (*V*_0_) was measured in a glass cylinder and after the addition of an excess of toluene the cylinder was sealed and the swollen polymer volume was recorded after 24 h (*V*_sw_). The swelling ratio was calculated according to Equation 1.

(1) $$Sr=\frac{V_{sw}}{V_{0}}$$

The surface area of the swollen polymers (*A*_sw_) was approximated using the assumption that the polymer particles are spherical. *A*_sw_ was calculated according to Equation 2.

(2) $$A_{sw}=A_{0}\cdot {Sr}^{2/3}$$

where *A*_0_ is the dry polymer BET surface area presented in Table 1.

## 4. Conclusions

The choice of methacrylic acid–ethylene dimethacrylate co-polymer composition influences both the morphology and warfarin binding (specific and non-specific) to the materials studied. A clear optimum was observed with respect to selective binding (MIP3/REF3). Lower degrees of cross-linking afforded greater capacities (relative to surface areas), though this binding was essentially non-specific in nature. Higher degrees of cross-linking (higher relative MAA concentrations) yielded lower selectivity. The distinct differences in the performance of materials as a function of morphology indicate that the manipulation of the degree of cross-linking should be more carefully considered in molecularly imprinted polymer design. As MAA-EDMA co-polymers are still today the most frequently reported MIPs, we believe that the results described here should be considered in conjunction with the design of new MIPs using these monomers, and even in the reappraisal of previous studies.

## Figures and Tables

**Table 1:** Polymer compositions.

Components	MIP1	MIP2	MIP3	MIP4	MIP5	REF1	REF2	REF3	REF4	REF5
Warfarin (mmol)	1.39	1.39	1.39	1.39	1.39					
MAA (mmol)	2.78	6.95	16.68	27.80	55.60	2.78	6.95	16.68	27.80	55.60
EDMA (mmol)	76.5	76.5	76.5	76.5	76.5	76.5	76.5	76.5	76.5	76.45
CHCl_3_ (mL)	23.5	24.0	25.3	26.8	30.6	23.5	24.0	25.3	26.8	30.6
AIBN (mmol)	2.02	2.08	2.20	2.35	2.71	2.02	2.08	2.20	2.35	2.71
EDMA:MAA ratio	27.5	11.0	4.6	2.8	1.4	27.5	11.0	4.6	2.8	1.4

**Table 2:** Polymer pore size distributions.

Polymer	Average pore size (nm)	

	peak 1	peak 2
MIP3	2.1	3.9
MIP4	3.6	22
MIP5	3.4	100

REF3	3.6	15
REF4	3.6	37
REF5	3.4	114

**Table 3:** Surface areas, rebinding data and swelling ratios.

Polymer	Surface area (m^2^/g) (r) a		PC_50_ (mg/mL) b	Sr c

	BET	Langmuir		
MIP1	35.4 ± 1.8 [0.994]	51.3 ± 2.2 [0.996]	13.2	2.50
MIP2	3.6 ± 0.5 [0.962]	5.8 ± 1.3 [0.917]	12.4	2.65
MIP3	73.5 ± 1.2 [0.999]	102.0 ± 3.1 [0.998]	7.3	2.33
MIP4	118.4 ± 0.6 [1.000]	163.5 ± 4.3 [0.999]	4.7	1.60
MIP5	50.0 ± 0.2 [1.000]	69.5 ± 2.3 [0.998]	12.7	1.30

REF1	2.3 ± 0.1 [0.999]	3.0 ± 0.0 [1.000]	13.5	2.40
REF2	2.6 ± 0.1 [0.996]	3.6 ± 0.2 [0.992]	11.9	2.60
REF3	238.3 ± 2.5 [1.000]	329.9 ± 8.4 [0.999]	9.0	2.00
REF4	169.1 ± 1.2 [1.000]	233.4 ± 5.7 [0.999]	7.1	1.35
REF5	83.3 ± 0.4 [1.000]	116.1 ± 3.1 [0.999]	9.9	1.30

a Areas presented as mean ± standard deviation from linear regression using the BET or the Langmuir methods where the resultant correlation coefficient is presented as r.

b Concentration of polymer required for 50% binding of template.

c Swelling ratio calculated using Equation 1.

## References

[1] Wulff G. (1984). Molecular imprinting. Ann. N. Y. Acad. Sci.

[2] Mosbach K., Ramström O. (1996). The emerging technique of molecular imprinting and its future impact on biotechnology. Biotechnology.

[3] Sellergren B. (2001). Molecularly Imprinted Polymers: Man-Made Mimics of Antibodies and Their Applications in Analytical Chemistry.

[4] Alexander C., Andersson H.S., Andersson L.I., Ansell R.J., Kirsch N., Nicholls I.A., O’Mahony J., Whitcombe M.J. (2006). Molecular imprinting science and technology: A survey of the literature for the years up to and including 2003. J. Mol. Recognit.

[5] Sellergren B., Shea K.J. (1993). Influence of polymer morphology on the ability of imprinted network polymers to resolve enantiomers. J. Chromatogr. A.

[6] Nicholls I. (1995). Thermodynamic considerations for the design of and ligand recognition by molecularly imprinted polymers. Chem. Lett.

[7] Wu X., Carroll W.R., Shimizu K.D. (2008). Stochastic lattice model simulations of molecularly imprinted polymers. Chem. Mater.

[8] Baggiani C., Giovannoli C., Anfossi L., Passini C., Baravalle P., Giraudi G. (2012). A Connection between the binding properties of imprinted and nonimprinted polymers: A change of perspective in molecular imprinting. J. Am. Chem. Soc.

[9] Nicholls I.A., Piletsky S.A., Chen B., Chianella I., Turner A.P.F., Yan M., Ramström O. (2004). Thermodynamic Considerations and The Use of Molecular Modeling as a Tool for Predicting MIP Performance. Molecularly Imprinted Materials.

[10] Karim K., Breton F., Rouillon R., Piletska E.V., Guerreiro A., Chianella I., Piletsky S.A. (2005). How to find effective functional monomers for effective molecularly imprinted polymers?. Adv. Drug Deliv. Rev.

[11] Nicholls I.A., Andersson H.S., Charlton C., Henschel H., Karlsson B.C.G., Karlsson J.G., O’Mahony J., Rosengren A.M., Rosengren K.J., Wikman S. (2009). Theoretical and computational strategies for rational molecularly imprinted polymer design. Biosens. Bioelectron.

[12] Nicholls I., Andersson H., Golker K., Henschel H., Karlsson B., Olsson G., Rosengren A., Shoravi S., Suriyanarayanan S., Wiklander J. (2011). Rational design of biomimetic molecularly imprinted materials: Theoretical and computational strategies for guiding nanoscale structured polymer development. Anal. Bioanal. Chem.

[13] Sajonz P., Kele M., Zhong G., Sellergren B., Guiochon G. (1998). Study of the thermodynamics and mass transfer kinetics of two enantiomers on a polymeric imprinted stationary phase. J. Chromatogr. A.

[14] Chen Y., Kele M., Sajonz P., Sellergren B., Guiochon G. (1999). Influence of thermal annealing on the thermodynamic and mass-transfer kinetic properties of d- and l-Phenylalanine anilide on imprinted polymeric stationary phases. Anal. Chem.

[15] Piletska E.V., Piletsky S.A. (2010). Size matters: Influence of the size of nanoparticles on their interactions with ligands immobilized on the solid surface. Langmuir.

[16] Karlsson B.C.G., Rosengren A.M., Näslund I., Andersson P.O., Nicholls I.A. (2010). Synthetic human albumin Sudlow I binding site mimics. J. Med. Chem.

[17] Li T., Chang C.-Y., Jin D.-Y., Lin P.-J., Khvorova A., Stafford D.W. (2004). Identification of the gene for vitamin K epoxide reductase. Nature.

[18] Rost S., Fregin A., Ivaskevicius V., Conzelmann E., Hortnagel K., Pelz H.-J., Lappegard K., Seifried E., Scharrer I., Tuddenham E.G.D. (2004). Mutations in VKORC1 cause warfarin resistance and multiple coagulation factor deficiency type 2. Nature.

[19] Karlsson B.C.G., Rosengren A.M., Andersson P.O., Nicholls I.A. (2007). The spectrophysics of warfarin: Implications for protein binding. J. Phys. Chem. B.

[20] Karlsson B.C.G., Rosengren A.M., Andersson P.O., Nicholls I.A. (2009). Molecular insights on the two fluorescence lifetimes displayed by warfarin from fluorescence anisotropy and molecular dynamics studies. J. Phys. Chem. B.

[21] Rosengren A.M., Karlsson B.C.G., Näslund I., Andersson P.O., Nicholls I.A. (2011). *In situ* detection of warfarin using time-correlated single-photon counting. Biochem. Bioph. Res. Commun.

[22] Rosengren A.M., Karlsson B.C.G., Nicholls I.A. (2012). Monitoring the distribution of warfarin in blood plasma. ACS Med. Chem. Lett.

[23] He M., Korzekwa K.R., Jones J.P., Rettie A.E., Trager W.F. (1999). Structural forms of phenprocoumon and warfarin that are metabolized at the active site of CYP2C9. Arch. Biochem. Biophys.

[24] Valente E.J., Lingafelter E.C., Porter W.R., Trager W.F. (1977). Structure of warfarin in solution. J. Med. Chem.

[25] Nicholls I.A., Karlsson B.C.G., Rosengren A.M., Henschel H. (2010). Warfarin: An environment-dependent switchable molecular probe. J. Mol. Recognit.

[26] Rosengren A.M., Karlsson B.C.G. (2011). Spectroscopic evidence for the presence of the cyclic hemiketal form of warfarin in aqeous solution: Consequences for bioavailability. Biochem. Biophys. Res. Commun.

[27] Lange H. (1986). Determination of the degree of swelling and cross-linking of extremely small polymer gel quantities by analytical ultracentrifugation. Colloid Polym. Sci.

[28] Brunauer S., Emmett P.H., Teller E. (1938). Adsorption of gases in multimolecular layers. J. Am. Chem. Soc.

[29] Langmuir I. (1916). The constitution and fundamental properties of solids and liquids. Part I. Solids. J. Am. Chem. Soc.

[30] Gregg S.J., Sing K.S.W. (1982). Adsorption, Surface and Porosity.

[31] Barrett E.P., Joyner L.G., Halenda P.P. (1951). The determination of pore volume and area distributions in porous substances. I. Computations from nitrogen isotherms. J. Am. Chem. Soc.

